# Mdivi-1 attenuates oxidative stress and exerts vascular protection in ischemic/hypoxic injury by a mechanism independent of Drp1 GTPase activity

**DOI:** 10.1016/j.redox.2020.101706

**Published:** 2020-08-29

**Authors:** Chenyang Duan, Li Wang, Jie Zhang, Xinming Xiang, Yue Wu, Zisen Zhang, Qinghui Li, Kunlun Tian, Mingying Xue, Liangming Liu, Tao Li

**Affiliations:** State Key Laboratory of Trauma, Burns and Combined Injury, Second Department of Research Institute of Surgery, Daping Hospital, Army Medical University, Chongqing, 400042, PR China

**Keywords:** Mdivi-1, Ischemic/hypoxic injury, Drp1, Mitochondrial fission, Oxidative stress, Nrf2, ALT, alanine aminotransferase, AST, aspartate aminotransferase, BUN, blood urea nitrogen, CREA, creatinine, Drp1, dynamin-related protein 1, LR, Lactated Ringer (solution), MAP, mean artery pressure, Mdivi-1, Mitochondrial division inhibitor 1, DMSO, dimethyl sulfoxide, Nrf2, NF-E2-related factor 2, SMA, superior mesenteric artery, VSMC, vascular smooth muscle cell

## Abstract

Vascular dysfunctions such as vascular hyporeactivity following ischemic/hypoxic injury are a major cause of death in injured patients. In this study, we showed that treatment with mitochondrial division inhibitor 1 (Mdivi-1), a selective inhibitor of dynamin-related protein 1 (Drp1), significantly improved vascular reactivity in ischemic rats by attenuating oxidative stress. The antioxidative effects of Mdivi-1 were relatively Drp1-independent, and possibly due to an increase in the levels of the antioxidant enzymes, SOD1 and catalase, as well as to enhanced Nrf2 expression. In addition, we found that while Mdivi-1 had little effect on Drp1 GTPase activity in vascular smooth muscle cells, it inhibited hypoxia-induced Drp1 phosphorylation at Ser-616, reducing excessive mitochondrial fission and slightly enhancing mitochondrial fusion. These effects possibly contributed to vascular protection at an early stage of ischemic/hypoxic injury. Finally, Mdivi-1 stabilized hemodynamics, increased vital organ perfusion, and improved rat survival after ischemic/hypoxic injury, proving a promising therapeutic agent for ischemic/hypoxic injury.

## Highlights

• Mdivi-1 improved vascular contractility in ischemic rats.
• Mdivi-1 attenuated hypoxia-induced oxidative stress and mitochondrial changes.
• Drp1 recruitment to mitochondria, not GTPase activity, involved in Mdivi-1 effects.
• Mdivi-1 has therapeutic potential against ischemic injury.

## 1 Introduction

Ischemia or hemorrhage refers to a sudden decrease in blood volume and pressure caused by rupture of blood vessels, which may induce anoxic necrosis of multiple organs or even death [1]. Although treatment of ischemic patients has recently improved owing to fluid resuscitation and correction of acidosis and coagulopathy [2], vascular dysfunctions such as vascular hyporeactivity following ischemia are still a major cause of death in these subjects [3]. Vascular hyporeactivity is a severe vascular dysfunction characterized by reduced vascular response to vasoconstrictors, such as norepinephrine (NE), in the treatment of ischemic/hypoxic injury. Our previous studies have demonstrated that enhancing vascular reactivity can be beneficial for the survival of ischemic patients and their vital organ functions [3,4]. Thus, a better comprehension of the mechanisms underlying the pathogenesis of ischemic/hypoxic injury, as well as the development of novel treatments for patients with vascular hyporeactivity remain major research priorities.

We previously demonstrated that systemic treatments aimed at the control of mitochondrial homeostasis, including mitochondrial quantity and mitochondrial functions, may promote cell survival and functional recovery in ischemic/hypoxic injury [[5], [6], [7]]. As one of the early damaged organelles in ischemic/hypoxic injury, the mitochondrion plays a central role in cell material and energy metabolism. When the mitochondrial respiratory chain is damaged, the accumulation of ROS in mitochondria leads to oxidative stress and, eventually, mitochondrial dysfunction, which is a major contributor to organ injury (e.g., liver, kidney) [8]. Dynamin-related protein 1 (Drp1) is an important regulator of mitochondrial fission and affects the quantity of mitochondria. We have recently found that Drp1 plays an important role in the regulation of mitochondrial homeostasis after ischemia or hypoxia in various tissues [[5], [6], [7]]. Since ischemic/hypoxic injury causes aberrant mitochondrial fission and/or disrupted membrane integrity, Drp1 is a candidate drug target for the treatment of this condition.

Mitochondrial division inhibitor 1 (Mdivi-1) is a small-molecule inhibitor of Drp1 GTPase activity [9] and exerts therapeutic effects in several conditions, including acute myocardial infarction [10], sepsis [11], and neurodegenerative diseases [12]. However, a recent study indicated that the effects of Mdivi-1 on mitochondria are not dependent on Drp1^13^. To date, the exact role of Drp1 in Mdivi-1 vascular protection after ischemic/hypoxic injury is still unclear.

We hypothesized that Mdivi-1 vascular protection against ischemia is related to its positive action on mitochondrial homeostasis, as well as to its ability to attenuate oxidative stress, possibly also involving a Drp1-independent mechanism. To test this hypothesis, we examined the effects of Mdivi-1 on vascular contraction and vital organ perfusion, and investigated the possible involvement of oxidative stress and Drp1 GTPase activity in Mdivi-1-induced effects. To this end, ischemic rats obtained by 50% total hemorrhage, *Drp1* knockdown mice, as well as an in vitro model based on hypoxia-treated vascular smooth muscles cells (VSMCs), were employed.

## 2 Materials and methods

### 2.1 Materials

Mdivi-1 (Cat.No.3982) was purchased from TOCRIS (Minneapolis, MN, USA) and stored at −20 °C. The molecular weight is 353.22.50 mg Mdivi-1 powder was dissolved in 2 ml DMSO and the prepared concentration was 25 mg/ml. The *in vivo* operating concentration was 1 mg/kg on rats or mice model and the in vitro operating concentration was 50 μM on vascular smooth muscle cells (VSMCs) model. Antibodies for Drp1, Nrf2, β-actin, Tubulin, Lamin B1 and COX IV were purchased from Abcam (Cambridge, MA, USA). Brusatol, the Nrf2 inhibitor, was purchased from JONLN (Shanghai, CHINA) and incubated at 5 μmol/L for 30min prior to Mdivi-1 in vitro. GFP-tagged Adenoviral vectors for Drp1 deletion (GTP-Ad-shDrp1) and Ad-shDrp1 were generated by Obio Technology (Shanghai, CHINA). Its interference effects on Drp1 has been reported in our previous study [6]. The Drp1 S616A mutation plasmid (S616A) and its blank control (Vector) was constructed by Genechem Biotechnology (Shanghai, CHINA). MitoTracker Deep Red and MitoSOX Red were purchased from Invitrogen (Carlsbad, CA, USA). The ATP detection kit, ROS fluorescent probes, MDA assay kit and superoxide assay kit for Catalase and SOD1 were purchased from Beyotime Biotechnology (Shanghai, CHINA). Oxidative stress defense Western blot Cocktail and antibodies for related markers including Catalase, SOD1 and TRX were purchased from Abcam (Cambridge, MA, USA). The DHE (Dihydroethidium) assay kit was purchased from Abcam (Cambridge, MA, USA). The mitochondria isolation kit for muscle tissues and Minute (TM) cytoplasmic and nuclear fractionation kit were purchased from Invent Biotechnology (Beijing, CHINA). All other chemicals were purchased from Sigma unless specifically mentioned otherwise.

### 2.2 Animal preparation and experimental protocol

Four hundred and eighty 8-week-old Sprague-Dawley (SD) male rats (200–220 g) were purchased from the Research Institute of Surgery, Army Medical University (Chongqing, China) and randomly divided into five groups: sham-operated group (Sham group), ischemia caused by 50% of total hemorrhage group (Ischemia group), conventional-treated group with Lactated Ringer (LR) solution (LR group), conventional-treated group combined with Mdivi-1 solvent dimethyl sulfoxide (DMSO) (DMSO group), and conventional treatment combined with Mdivi-1 (1 mg/kg) group (Mdivi-1 group).

For the Ischemia group, rats were anaesthetized with sodium pentobarbital (initial dosage, 30 mg/kg) and placed on a warmed plate to maintain the body temperature at 37 °C. Aseptic techniques were adopted for all surgical procedures. The right carotid artery and vein were catheterized for monitoring hemodynamics. Right femoral artery was catheterized with polyethylene catheters filled with heparinized saline for bleeding 50% of total blood volume (≈7% of weight) and maintained for 4 h [14]. For the Sham group, rats were operated on as mentioned above, except for the bleeding operation. After the ischemia model was established, the rats in LR group were infused with two volumes, of blood loss, of LR solution through the right femoral vein within 30 min. The rats in Mdivi-1 group received a continuous infusion of Mdivi-1 (1 mg/kg) combined with LR resuscitation, while the rats in DMSO group only received the corresponding dose of DMSO used for Mdivi-1 administration in the process of LR resuscitation. After resuscitation, animal survival, hemodynamics, vital organ perfusion and functions, as well as mitochondrial homeostasis were observed. At the end of the experiments, rats were euthanized with a lethal dose of sodium pentobarbital (100 mg/kg, iv).

Fifty specific-pathogen-free C57BL/6 male mice (~8 weeks old, 18–25 g) and Fifty Drp1 knockdown (Drp1 +/−) male mice (~8 weeks old, 20–23 g) were purchased from Shanghai Model Organisms Center, Inc (Shanghai, CHINA), and housed in the animal facility of Army Medical University (Chongqing, CHINA) for a week prior to the start of the experiments. The knockdown effects were proven in our previous studies [6,7]. The model preparation and treatment procedures in mice were the same as in rats. All procedures were approved by the Laboratory Animal Welfare and Ethics Committee of Army Medical University (No.AMUWEC20171288). The investigation conformed to the protocols in the Guide for the Care and Use of Laboratory Animals (National Institutes of Health, Publication No. 85-23, Revised 1996).

### 2.3 Cell culture and hypoxia treatment

Vascular smooth muscle cells (VSMCs) were obtained from the superior mesenteric arteries (SMAs) of SD rats using an explant technique, as previously described [15]. VSMCs were cultured in Dulbecco-modified Eagle medium (DMEM)-F12 (Gibco, NY, USA) supplemented with 10% fetal bovine serum (FBS) (Hyclone, Logan, UT, USA) and 1% antibiotics. The third-to-fifth passage cells were used in the present study.

For hypoxic experiments, VSMCs were cultured in low-glucose DMEM (1000 mg/L d-glucose) without FBS, and transferred into a hypoxia culture compartment (MIC-101, Billups-Rothenberg, Del Mar, CA) equilibrated with 95% N_2_ and 5% CO_2_, in which the estimated oxygen concentration was less than 0.2%, and thereafter incubated for 4 h under hypoxic conditions [6]. After hypoxia treatment, VSMCs were treated with 50 μM Mdivi-1 in the Mdivi-1 group, and with the corresponding dose of DMSO in the DMSO group.

### 2.4 Measurements of vascular reactivity and VSMC contraction

The vascular reactivity of SMA tissues to norepinephrine (NE) was observed using an isolated organ perfusion system (Scientific Instruments, Barcelona, Spain). Briefly, each SMA was cut into 1–2 mm ring, which was suspended between a force transducer and then immersed into an isolated organ chamber (Scientific Instruments, Barcelona, Spain) containing 5 ml K–H solution. The vascular constriction reactivity of the artery rings to different concentrations of NE (10^−9^, 10^−8^, 10^−7^, 10^−6^ and 10^−5^ mol/L) were recorded by a Power Lab System via a force transducer [4].

To direct observe vasocontraction of SMAs *in vivo*, the abdomen was opened via a midline incision (20–30 mm in length). The ileocecal portion of the mesentery was gently exteriorized and mounted on a transparent plastic stage. Single unbranched arterioles without obvious bends (diameter range from 30 μm to 50 μm, length of approximately 200 μm) were selected for observation. The mesentery was kept warm and moist by continuous superfusion with saline solution at 37 °C. Following equilibrium for 10min, 100 μl NE (10^−7^, 10^−6^, 10^−5^, 10^−4^ and 10^−3^ mol/L) were added to the mesenteric surface to contract the arterioles and the alterations in diameter were recorded with a video camera (Olympus, DP21) and measured with Image J software [16].

As for the contraction ability of adult acute-separated VSMCs, the isolated SMA tissues were cut into 1 mm^2^ pieces to shakily enzyme with digestive solution (0.02 g type II collagenase, 0.08 g papain, 0.02 g bovine serum albumin (BSA), 0.015 g dithiothreitol (DTT) in 10 ml PS solution) at 37 °C for 20min. The digestion was stopped with low-calcium physiological saline solution (Ca^2+^ 0.02 mmol/L). The undigested tissues were filtered out and centrifugated for 1min. The supernatant was discarded and the acute-separated VMSCs were plated in confocal chamber and stimulated by NE (10^−3^ mol/L). The range of contraction area represented the constriction ability of VSMCs. Similar procedures were also operated on cultured VSMCs in vitro.

### 2.5 Mitochondrial DNA (mtDNA) copy number detection

The mtDNA copy number detection was taken to reflect the amount of mitochondria. Total DNA was extracted from SMA tissues of rats. The amount of mitochondrial DNA relative to nuclear DNA was determined by quantitative real-time PCR using primers for Nd2 (mitochondrial genome, Rn03296765_s1; Invitrogen) and GAPDH (nuclear genome, Rn01775763_g1; Invitrogen). Relative mtDNA copy number was calculated based on the threshold cycle (Ct) as 2^−Δ(ΔCt)^, where ΔCt = Ct_Nd2_ – Ct_GAPDH_, and Δ(ΔCt) = ΔCt _sample_ – ΔCt _control_ [17].

### 2.6 Mitochondrial dynamics observation

Mitochondria in VSMCs were labeled by MitoTracker Deep Red (100 nM) at 37 °C for 30min, and observed by confocal microscopy (Leica TCS SP5, Wetzlar, Germany) with a 60 × 1.3 NA oil-immersion objective for time-lapse recording mitochondrial dynamics every 15min, including mitochondrial fission and mitochondrial fusion events. The accumulated fission/fusion events were calculated and the slope of linear regression represented the real-time fission/fusion rate at each time point. The mitochondrial fluorescence was excited by 633 nm laser and emission collected at 655-670655 nm.

### 2.7 GTPase activity assay

The recombinant Drp1 was expressed and purified as previously described [13]. Plasmid encoding Drp1 was transformed into *Escherichia coli* BL21 (DE3). The protein expression induction, the affinity chromatography isolation of His6-tagged Drp1 and the purification of concentrated Drp1 protein were assisted by Genechem Biotechnology (Shanghai, CHINA) and frozen in dry ice and ethanol bath at −80 °C. No loss of activity compared to fresh protein was observed if frozen aliquots were used within 12 h of thawing. Drp1 activity was measured at 0–100 μM Mdivi-1 under normal and hypoxia conditions using a GTPase assay. GTPase activity was determined in a 96-well format using 150 μl of GTPase reaction buffer (25 mM HEPES, 25 mM PIPES, 7.5 mM KCl, 5 mM MgCl2, 1 mM phosphoenolpyruvate, 20 units/ml pyruvate kinase/lactate dehydrogenase, 600 μM NADH, pH 7.0), which was placed into a 96-well Corning Costar flat, clear bottom plate (Sigma-Aldrich, St. Louis, MO, USA). Reactions were run using 5 μM Drp1, 150 mM NaCl, and 5% DMSO at indicated GTP and Mdivi-1 concentrations. The oxidation of NADH was measured at A340 for 40 min at 25 °C using a Molecular Devices (Sunnyvale, CA, USA) Flexstation 3 Multi-Detection Reader with Integrated Fluid Transfer. The non-hydrolysable GTP analogue GTP-γ-S was used as the negative control. Global analysis of the data was fit using Igor Pro to the following equation that models uncompetitive inhibition: Vo=(Vmax × [GTP])/(KM+(1+(I/Ki)) × [GTP]) [18]. Data from three independent preparations of enzyme were reported with standard errors of the mean and were representative of similar observations conducted with multiple preparations at different concentrations of Drp1.

### 2.8 Molecular docking of Mdivi-1 to Drp1

The molecular docking of Mdivi-1 to Drp1 were conducted in Yinfo Cloud Platform (http://cloud.yinfotek.com/) [19]. The chemical structure of the small molecule Mdivi-1 was drawn by JSME and converted to 3D structure with energy minimization in MMFF94 force field. The crystal/NMR structure of Drp1 protein (PDB code: 5WP9) was automatically downloaded from the RCSB Protein Data Bank (http://www.rcsb.org/). All redundant atoms except chain A were deleted and then the protein structure carefully treated in several steps including residue repairing, protonation, and partial charges assignment in AMBER ff14SB force field. The DMS tool was employed to build molecular surface of the receptor using a probe atom with a 1.4 Å radius. The binding pocket was defined by the residues and spheres were generated filling the site by employing the Sphgen module in UCSF Chimera. Grids necessary for rapid score evaluation were created by the Grid module. DOCK 6.7 ^20^ program was utilized to execute semi-flexible docking and output poses were evaluated by the Grid scoring function.

### 2.9 Immunofluorescence staining

VSMCs were plated in confocal chamber and incubated with MitoTracker Deep Red (100 nM for 30min, 37 °C). After washing twice in 1X phosphate-buffered saline (PBS), VSMCs were fixed in a 4% paraformaldehyde solution for 20 min at room temperature. Cells were permeabilized with 0.1% Triton-x 100 in 1X PBS for 5 min at room temperature. Cells were then blocked in a 5% BSA solution for 1 h at room temperature, washed and incubated overnight at 4 °C with primary antibodies against Drp1, Nrf2 or CATA. Cells were then washed in PBS plus 0.1% Tween-20 and incubated with corresponding fluorophore-conjugated mouse or rabbit secondary antibodies (Invitrogen, Carlsbad, CA, USA) for 1 h at room temperature. Cells were washed as before with a final wash in 1X PBS alone and incubated with DAPI (BD Biosciences, Franklin Lakes, NJ, USA) (1:50) for 5 min at room temperature. Immunofluorescence was visualized using confocal laser-scanning microscopy (Leica SP5, Germany) [7].

### 2.10 Western blotting

Subcellular fractionation, including cytoplasmic, mitochondrial and nuclear fractions, were isolated based on the kit instructions (Invent SC-003/NM-038; Beijing, CHINA). β-actin, Tubulin, COX IV and Lamin B1 were respectively used as interior references of total, cytoplasmic, mitochondrial and nuclear fractions. Fractioned proteins and total proteins were used for immunoblotting analyses with indicated antibodies as previously described [6]. The intensity of the bands was analyzed by Quantity One V 4.62 Software (Bio-Rad, Life Science, California, USA).

### 2.11 Measurement of intracellular ROS and mitochondrial ROS

The measurement of intracellular reactive oxygen species (ROS), including 2′,7′-Dichlorodihydrofluorescein diacetate (DCFH-DA) detection and Dihydroethidium (DHE) detection, in SMA tissues or VSMCs were undertaken based on the kit instructions as previously described [13,20]. Briefly, SMAs or VSMCs were incubated with 10 μM DCFH-DA or DHE for 30 min at 37 °C. DCFH-DA or DHE fluorescence was detected at 488 nm excitation and 501–563 nm emission by a confocal microscopy (Leica TCS SP5, Wetzlar, Germany) with a 5 × objective to SMA tissues and 40 × objective to VMSCs. Images for VSMCs were collected at 15min, 1 h and 2 h after 50 μM Mdivi-1 incubation in each group and the mean intensities of DCFH-DA fluorescence were calculated using Leica TCS software. The DHE fluorescence intensity (arbitrary units, A.U.) in SMA tissues from mice over time was analyzed in blinded fashion using Image J software and the rate of DHE change was represented by the slope of linear regression at each time point.

The measurement of mitochondrial ROS was based on the fluorescence results of MitoSOX live-cell imaging (5 μM for 10min, 37 °C). The MitoSOX red fluorescence of VSMCs in each group were detected at 580 nm excitation and 510 nm emission by the confocal microscopy at 2 h after 50 μM Mdivi-1 incubation. The mean intensities of MitoSOX Red fluorescence were calculated using Image J software.

### 2.12 Transmission electronic microscopy (TEM) imaging

The fresh SMA tissue samples from Drp1+/+ and Drp1 +/- mice were obtained and quickly fixed as previously described [6]. Samples were viewed and imaged with a H-7500 transmission electron microscope (TEM) (Hitachi, Japan). The mitochondrial numbers and the length of each mitochondrial individuals were calculated by Image J software.

### 2.13 Oxidative stress detection

The levels of oxidative stress biomarkers including malondialdehyde (MDA), catalase (CATA), superoxide dismutase 1 (SOD1) and thioredoxin (TRX) were detected with commercial assay kits (Beyotime; Shanghai, CHINA) according to the manufacturer's instructions. Oxidative stress defense western blotting was also undertaken to detect the ROS-dependent oxidative stress in each group.

### 2.14 Vital organ functions measurement

The rats underwent a laparotomy and the blood flow in the liver and kidney was measured by a laser speckle technique (PeriScan PIM3 System; Stockholm, Sweden). Blood sample was withdrawn for determination of liver and kidney functions, including aspartate aminotransferase (AST), alanine aminotransferase (ALT), blood urea nitrogen (BUN) and creatinine (CREA) by a biochemical analyzer (Beckman, Fullerton, CA), as previously described [21].

### 2.15 Statistics analysis

Most of the results were expressed as Means and Standard Deviations (SD). The statistics analysis of survival time was expressed as Means and Standard Error of Mean (SEM). Independent sample *t*-test was used for experiments with two groups. One-way analysis of variance (ANOVA) was used for experiments with more than two groups and followed by Tukey's post hoc analysis and SNK or LSD comparison. The survival analysis was calculated by Kaplan-Meier method using SPSS 17.0 (SPSS Inc., Chicago, IL, USA). P < 0.05 was considered statistically significant.

## 3 Results

### 3.1 Mdivi-1 improves vascular reactivity and VSMC contraction after ischemic/hypoxic injury

**Fig. 1:**
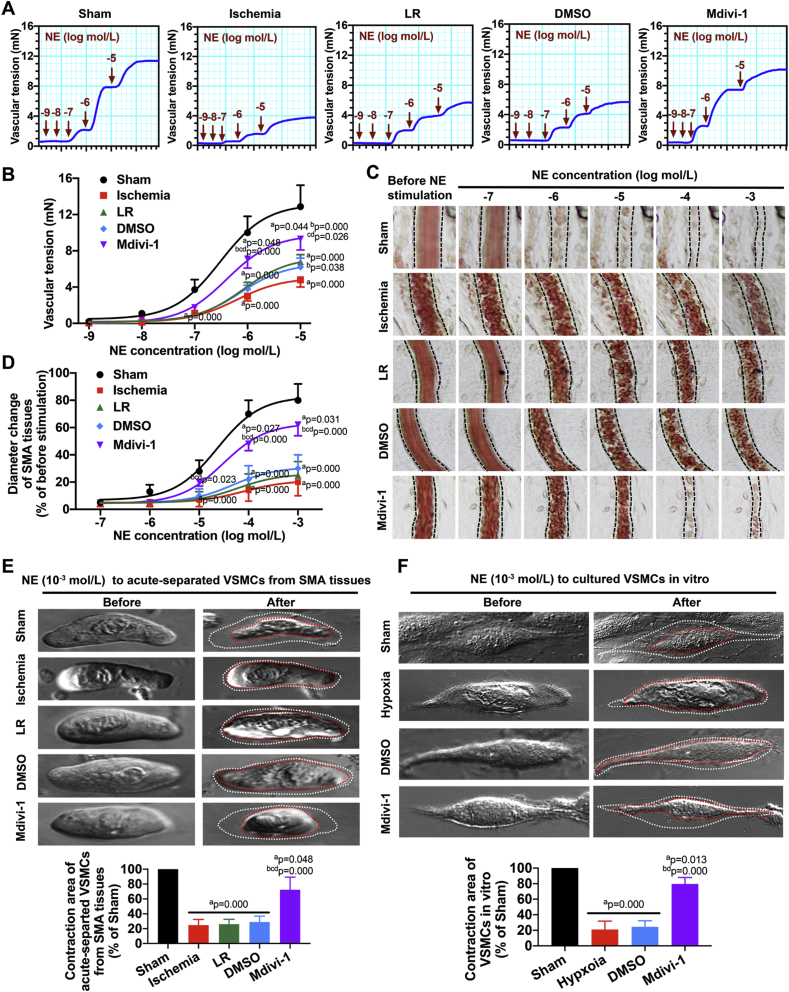
**Effects of Mdivi-1 on vascular functions in ischemic/hypoxic injury**. (A) Vascular tension curve of SMA to different concentrations of NE (10^−9^, 10^−8^, 10^−7^, 10^−6^ and 10^−5^ mol/L). (B) The statistics of vascular reactivity in SMA tissues to NE stimulation (n = 8 rats/group). (C) Alteration in SMA diameter to different concentrations of NE (10^−7^, 10^−6^, 10^−5^, 10^−4^ and 10^−3^ mol/L). The vascular boundaries were labeled in dotted line. (D) The statistics of diameter change in SMA to NE stimulation (n = 8 rats/group). (E–F) The contractility of acute-separated VSMCs (E, n = 8 rats/group) and cultured VSMCs (F, n = 16 cells/group) to 10^−3^ mol/L NE stimulation. The cell boundaries before NE stimulation were labeled in white dotted curve and the cell boundaries after NE stimulation were labeled in red dotted curve. The range of contraction areas in VSMCs were measured by Image J software. a: P < 0.05 compared with the Sham group at the same time point, b: P < 0.05 compared with the Ischemia group at the same time point, c: P < 0.05 compared with the LR group at the same time point, d: P < 0.05 compared with the DMSO group at the same time point. (For interpretation of the references to colour in this figure legend, the reader is referred to the Web version of this article.)

The maximum contractile activity of isolated SMAs in response to norepinephrine (NE) was 12.88 ± 2.36 mN in sham rats and significantly decreased to 5.31 ± 0.80 mN after ischemia (P < 0.05), as previously reported [4]. Mdivi-1 markedly restored the contraction of isolated SMAs, as compared to LR- and DMSO-treated rats (Fig. 1A–B) (P < 0.05). A protective effect of Mdivi-1 on vascular reactivity after ischemia was also observed *in vivo*. In sham rats, the SMA diameter significantly decreased by 80% after stimulation with 10^−3^ mol/L NE, while no significant changes were observed in ischemic, LR-, and DMSO-treated rats regardless of NE concentration (P < 0.05). In Mdivi-1-treated rats, vascular contraction in response to NE was restored, and the SMA diameter decreased by up to 62% after NE stimulation (P < 0.05) (Fig. 1C–D). Next, the contractility was analyzed in VSMCs that had been acute-separated from SMA tissues. While LR resuscitation after ischemia failed to restore VSMC contractile response to NE, Mdivi-1 infusion significantly improved the contractility by 46.3% with respect to VSMCs from LR-treated rats (P < 0.05) (Fig. 1E). A similar trend was also observed in hypoxia-treated VSMCs after incubation with Mdivi-1 (Fig. 1F).

### 3.2 Mdivi-1 exerts beneficial effects on VSMC mitochondrial dynamics at early stages of ischemic/hypoxic injury

**Fig. 2:**
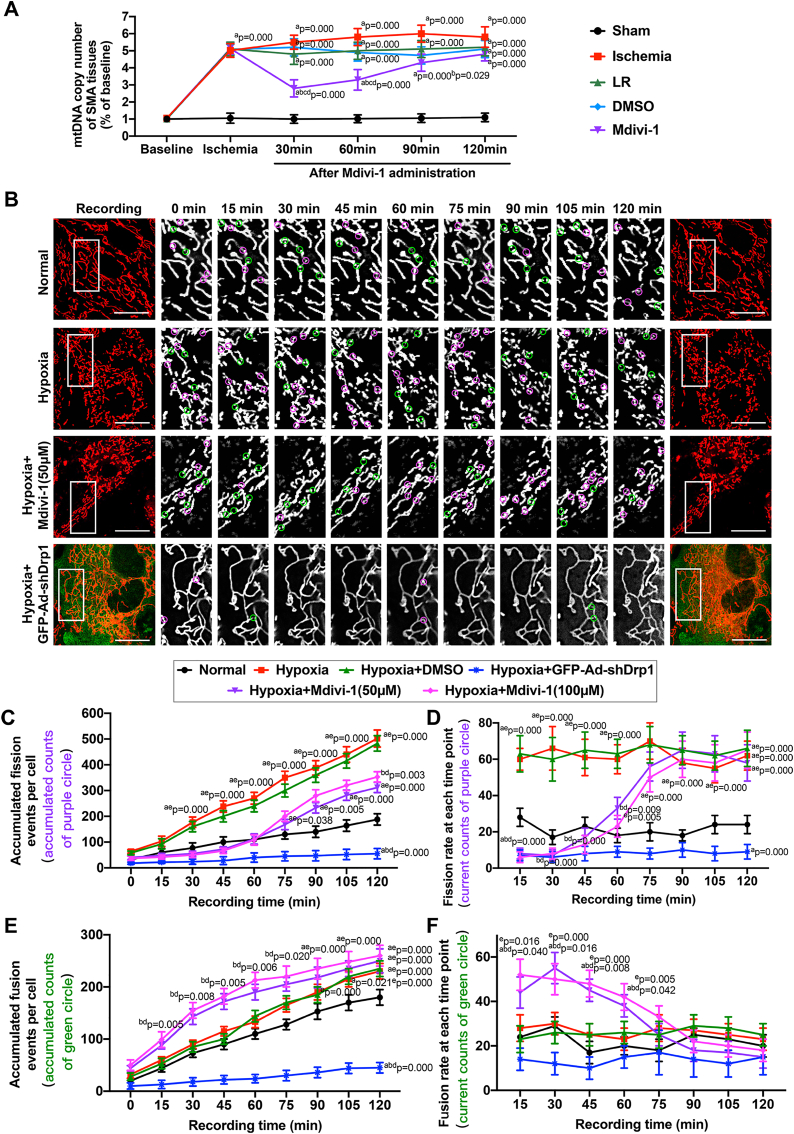
**Effects of Mdivi-1 on mitochondria dynamics in ischemic/hypoxic injury**. (A) mtDNA copy number in vascular tissues (n = 8 rats/time point in each group). (B) Time-lapse recording of mitochondrial dynamics, including fission and fusion events, for 120min (15min interval). Mitochondrial fission sites were circled in purple and mitochondrial fusion sites were circled in green (bar = 25 μm). (C–D) Accumulated fission events (C) and real-time fission rate (D) per cell after Mdivi-1 administration at concentration of 50 and 100 μM in hypoxic VSMCs (n = 16 cells/group). (E–F) Accumulated fusion events (E) and real-time fusion rate (F) per cell after Mdivi-1 administration at concentration of 50 and 100 μM in hypoxic VSMCs (n = 16 cells/group). a: P < 0.05 compared with Sham or Normal group at the same time point, b: P < 0.05 compared with Ischemia or Hypoxia group at the same time point, c: P < 0.05 compared with the LR group at the same time point, d: P < 0.05 compared with the DMSO or Hypoxia + DMSO group at the same time point, e: P < 0.05 compared with the Hypoxia + GFP-Ad-shDrp1 group at the same time point. (For interpretation of the references to colour in this figure legend, the reader is referred to the Web version of this article.)

In SMA tissues, the mtDNA copy number, which reflects the quantity of mitochondria, significantly increased by 4.02 times after ischemia (P < 0.05). Although infusion of LR alone slightly reduced the mtDNA levels in SMAs, the difference was not statistically significant (P > 0.05). Notably, early Mdivi-1 administration significantly decreased mtDNA copy number compared to LR- and DMSO-treated rats, especially at 30 min (P < 0.05). However, after 2 h, the mtDNA copy number reversely increased in the Mdivi-1 group (P > 0.05). The above *in vivo* results suggested that early administration of Mdivi-1 inhibited the ischemia-induced increase in the level of mtDNA (Fig. 2A).

To further investigate the effects of Mdivi-1 on mitochondrial dynamics in vitro, we monitored mitochondrial fission and fusion events in primary VSMCs from rat SMAs. Normal mitochondrial dynamics was observed under physiological conditions, while excessive mitochondrial fission and a slight compensatory increase in the rate of mitochondrial fusions were observed after induction of hypoxia (P < 0.05) (Fig. 2B). In VSMCs exposed to hypoxia, early administration of 50 μM Mdivi-1 (15–60 min) significantly inhibited mitochondrial fission, and restored a normal rate of fission (P < 0.05) (Fig. 2C–D). However, Mdivi-1 had no effect on mitochondrial fission at later times (90–120 min) compared with hypoxia or DMSO group, neither did a higher concentration of 100 μM Mdivi-1 cause a more profound effect at earlier time points (P > 0.05) (Fig. 2D). In addition, an increased rate of mitochondrial fusion was observed in VSMCs treated with 50 μM or 100 μM Mdivi-1, and the effect was statistically significant at early stages (15–60 min), as compared to hypoxia or DMSO group (P < 0.05) (Fig. 2E–F). However, the mitochondrial fusion rate was not significantly affected when Mdivi-1 was administered at 75–120 min after ischemia induction, regardless of the concentration of Mdivi-1 (P > 0.05) (Fig. 2F). The above results further indicated that early administration of Mdivi-1 may be beneficial for mitochondrial dynamics, resulting in inhibition of mitochondrial fission and stimulation of mitochondrial fusion in hypoxia-induced VSMCs, in line with the results of *in vivo* mtDNA analysis (Fig. 2A).

As expected, a robust inhibition of mitochondrial fission and a slight compensatory decrease of mitochondrial fusion were observed in hypoxia-induced VSMCs transfected with GFP-tagged Adenovirus Scramble Drp1 (GFP-Ad-shDrp1) (Fig. S1). However, the stable mitochondrial stasis induced by Ad-shDrp1 was clearly distinct from that of Mdivi-1 on mitochondrial dynamics in VSMCs exposed to hypoxia (P < 0.05) (Fig. 2B–F), suggesting that Mdivi-1 effects were not completely dependent on Drp1.

### 3.3 Mdivi-1 has little effect on Drp1 GTPase activity but inhibits hypoxia-induced Drp1 activation at Ser-616 and its translocation to mitochondria in VSMCs

**Fig. 3:**
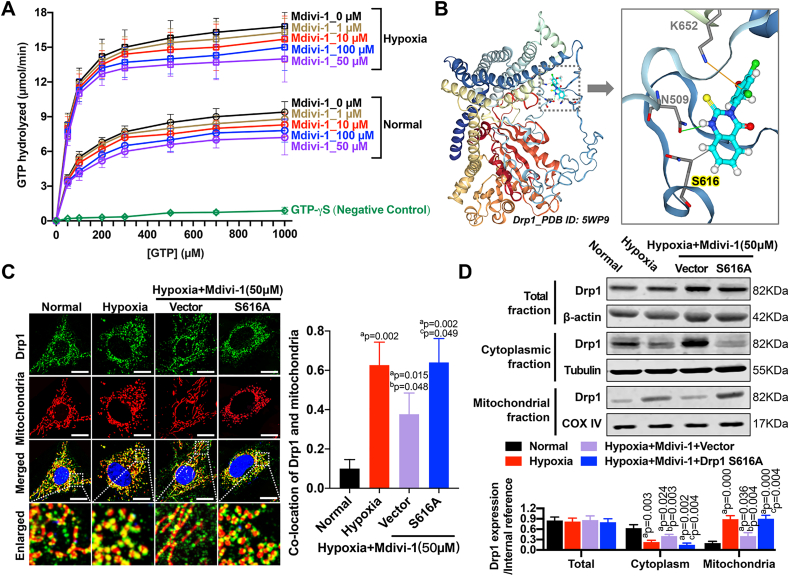
**Effects of Mdivi-1 on Drp1 translocation and its GTPase activity in hypoxia-treated VSMCs**. (A) Substrate kinetics of recombinant Drp1 to GTP hydrolysis was measured at 0–100 μM Mdivi-1 under normal and hypoxia conditions. The non-hydrolysable GTP analogue GTP-γ-S was used as the negative control. The data and SEM was for 3 independent preparations of Drp1. (B) Molecular docking of Mdivi-1 to Drp1. The PDB_5WP9 was used as the crystal/NMR structure of Drp1 protein. (C) Co-location of Drp1 and mitochondria in hypoxia-treated VSMCs after disturbing Mdivi-1 effect by Drp1 S616A mutation (n = 16 cells/group); (D) The relative protein expression of Drp1 in total, cytoplasmic and mitochondrial fractions of hypoxia-treated VSMCs after Drp1 S616A mutation. β-actin, Tubulin and COX IV were used as interior references of total, cytoplasmic and mitochondrial fractions. (n = 8 samples/group) a: P < 0.05 compared with Normal group, b: P < 0.05 compared with Hypoxia group, c: P < 0.05 compared with Hypoxia + Mdivi-1 (50 μM)+Vector group.

To verify whether Mdivi-1 directly antagonized mammalian Drp1 GTPase activity, recombinant Drp1 exhibiting assembly-stimulated GTPase activity [13,22] was employed. We found that the hydrolysis of Drp1-bound GTP was significantly increased after hypoxia. Notably, Mdivi-1 at various concentrations had negligible effects on Drp1 GTPase activity in VSMCs, under both normal and hypoxic conditions (Fig. 3A). As expected, strong inhibition of Drp1 GTPase activity was observed in the presence of non-hydrolysable GTP analogue, GTP-γ-S (Fig. 3A). Furthermore, molecular docking allowed us to predict a direct interaction between Mdivi-1 and Drp1 at Ser-616 (Fig. 3B). Notably, this interaction had some effects on mitochondrial proportion of Drp1 in hypoxia-treated VSMCs, and the localization of Drp1 in the mitochondria was significantly increased upon reversing Mdivi-1-inhibited Drp1 activity by S616A mutation (Fig. 3C). This was consistent with the variations in Drp1 expression in different cellular fractions as determined by western blotting. No changes in total Drp1 expression were detected in any of the groups (P > 0.05), while a marked and significant redistribution of Drp1 from the cytoplasmic to the mitochondrial fraction was observed after hypoxia (P < 0.05). Mdivi-1 partially suppressed hypoxia-induced Drp1 translocation to mitochondria in VSMCs, and this effect was abolished by the Drp1 S616A mutation (P < 0.05) (Fig. 3D). The above results indicated that, although Mdivi-1 had little effect on hypoxia-induced Drp1 GTPase activity in VSMCs, it affected Ser-616-dependent mitochondrial translocation of Drp1.

### 3.4 Mdivi-1 reduces ischemic/hypoxia-induced oxidative stress in a Drp1-independent manner

**Fig. 4:**
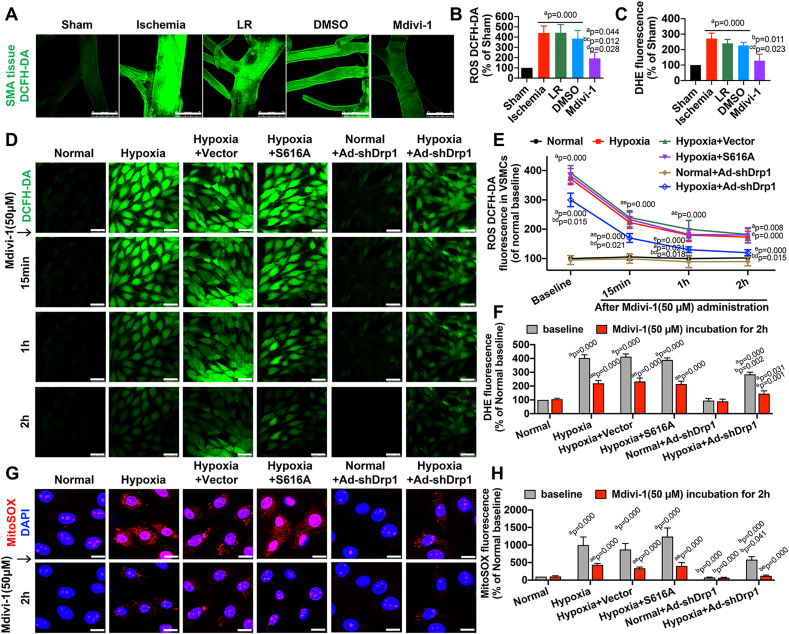
**Effects of Mdivi-1 on oxidative stress in ischemic/hypoxic injury**. (A) Confocal images showing intracellular ROS levels in SMA tissues (bar = 1 mm). (B) Quantitative data of the mean intensity of ROS DCFH-DA fluorescence in SMA tissues (n = 8 rats/group). (C) Quantitative data of the mean intensity of DHE fluorescence in SMA tissues (n = 8 rats/group). (D) Time-lapse recording of ROS DCFH-DA fluorescence in Mdivi-1 (50 μM)-incubated VSMCs at 15min, 1 h and 2 h (bar = 50 μm). (E) Quantitative data of ROS DCFH-DA fluorescence in VSMCs after treatment with Mdivi-1 (50 μM) (8 samples/group). (F) Quantitative data of DHE fluorescence in VSMCs after treatment with Mdivi-1 (50 μM) for 2 h (8 samples/group). (G) Mitochondrial ROS MitoSOX fluorescence in Mdivi-1 (50 μM)-incubated VSMCs at 2 h (bar = 25 μm). (H) Quantitative data of MitoSOX fluorescence in VSMCs after treatment with Mdivi-1 (50 μM) for 2 h (8 samples/group). a: P < 0.05 compared with Sham or Normal group at the same time point, b: P < 0.05 compared with Ischemia or Hypoxia group at the same time point, c: P < 0.05 compared with LR group at the same time point, d: P < 0.05 compared with DMSO or Hypoxia + Vector group at the same time point, e: P < 0.05 compared with the baseline of each group.

At the end of ischemia, the intracellular levels of ROS were significantly increased compared to normal rats, as determined by DCFH-DA assay in SMA tissues (P < 0.05). Mdivi-1 substantially prevented the ischemia-induced increase in ROS intracellular levels in SMAs (P < 0.05) (Fig. 4A–B). A similar trend was observed for ROS-dependent oxidative stress, based on detection of dihydroethidium (DHE) fluorescence (Fig. 4C). In VSMCs exposed to hypoxia for 4 h, a significant increase in DCFH-DA fluorescence was observed (P < 0.05). Treatment with Mdivi-1 (50 μM) significantly reduced ROS levels in hypoxic VSMCs in a time-dependent manner (P < 0.05), and further, S616A mutant did not affect DCFH-DA fluorescence at any time point (P > 0.05) (Fig. 4D–E), indicating that the effect of Mdivi-1 on oxidative stress was independent of Mdivi-1 effect on Ser-616-regulated Drp1 translocation. In VSMCs with Ad-shDrp1-driven low Drp1 expression, we observed a decrease in DCFH-DA fluorescence when compared with the baseline of the hypoxia group (P < 0.05); however, Mdivi-1 treatment could further inhibit the increase in ROS levels in Ad-shDrp1-transfected hypoxia-exposed VSMCs (P < 0.05) (Fig. 4D–E). Similar results were obtained after DHE labeling (Fig. 4F), and the trends of mitochondrial ROS assayed by MitoSOX were consistent with the variations in whole ROS levels observed after Mdivi-1 treatment (Fig. 4G–H). These results further suggested that the antioxidative effects of Mdivi-1 mainly focused on mitochondrial homeostasis dysfunction after ischemic/hypoxic injury, and the inhibitory effect exerted by Mdivi-1 on hypoxia-induced oxidative stress was, at least in part, independent of Drp1 function.

**Fig. 5:**
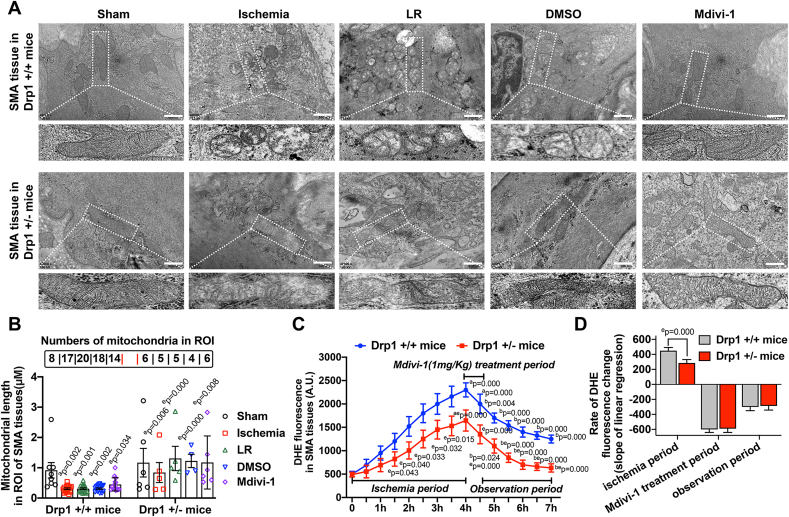
**Effects of Mdivi-1 on ischemia-induced oxidative stress in Drp1 knockdown mice**. (A) TEM images showing mitochondria in SMA tissues from Drp1 +/+ mice or Drp1 +/- mice. The enlarged images showing the inner cristae structures of mitochondria (bar = 500 nm). (B) Quantitative data in the length of each mitochondrial individuals in ROI (regions of interest) from SMA tissues. Numbers of mitochondria were labeled in the top of each group. (C) DHE fluorescence (arbitrary units, A.U.) of SMA tissues was measured in different periods (30min interval) in Drp1 +/+ or Drp1 +/- mice (8 mice/group). (D) Rate of DHE fluorescence change in Drp1 +/+ or Drp1 +/- mice. The rates were represented by the slope of linear regression in each period (8 mice/group). a: P < 0.05 compared with Sham group of the same genotype mice, b: P < 0.05 compared with Ischemia group of the same genotype mice, c: P < 0.05 compared with LR group of the same genotype mice, d: P < 0.05 compared with DMSO group of the same genotype mice, e: P < 0.05 compared with Drp1 +/+ mice at the same time point.

To further explore the involvement of Drp1 in the effects of Mdivi-1 on mitochondrial oxidative stress after ischemic/hypoxic injury, mitochondrial length, quantity, as well as the status of inner cristae structures were investigated in Drp1 knockdown mice (Drp1 +/−) after treatment with Mdivi-1 (Fig. 5A). After a 4-h ischemia and LR resuscitation, the mitochondrial length was significantly decreased, while the mitochondrial number was markedly increased (P < 0.05), as assessed by TEM (Fig. 5B). These effects were due to excessive Drp1-mediated mitochondrial fission, as we previously demonstrated [6]. Drp1 knockdown resulted in a significant elongation of mitochondria after ischemia, as compared to WT mice (Drp1 +/+) (P < 0.05). However, the inner cristae structures in WT mice mitochondria were significantly disorganized after ischemia and LR resuscitation, while Drp1 knockdown did not have a major impact on the inner cristae (see the enlarged images in Fig. 5A). After 2 h of Mdivi-1 treatment, the structure of mitochondrial cristae was better preserved than in the absence of Mdivi-1 in the DMSO group (P < 0.05) (Fig. 5B). Although a slight reversal in mitochondrial length and quantity were observed in ischemic mice after Mdivi-1 treatment, the differences were not statistically significant in either the WT or Drp1 knockdown mice (P > 0.05) (Fig. 5B). DHE staining showed that Drp1 knockdown partially inhibited ischemia-induced ROS production (Fig. 5C–D), which was consistent with our previous studies [6,7]. However, DHE fluorescence was significantly higher in SMAs of ischemic Drp1 knockdown mice compared to the baselines in normal condition (P < 0.05) (Fig. 5C), suggesting that ischemia-induced ROS enhancement was not entirely dependent on Drp1. In addition, Mdivi-1 significantly decreased DHE fluorescence in SMAs from both ischemia-treated WT and Drp1 knockdown mice (P < 0.05) (Fig. 5C). The above results suggested that, although Drp1 knockdown could slow down the increasing rate of ROS levels, it had no influence on the rate of ROS removal caused by Mdivi-1 (Fig. 5D), further confirming that the antioxidative effects of Mdivi-1 in VSMCs after ischemic/hypoxic injury were partially Drp1-independent.

### 3.5 Mdivi-1 attenuates oxidative stress by promoting the production of antioxidant enzymes and the expression of Nrf2 after ischemic/hypoxic injury

**Fig. 6:**
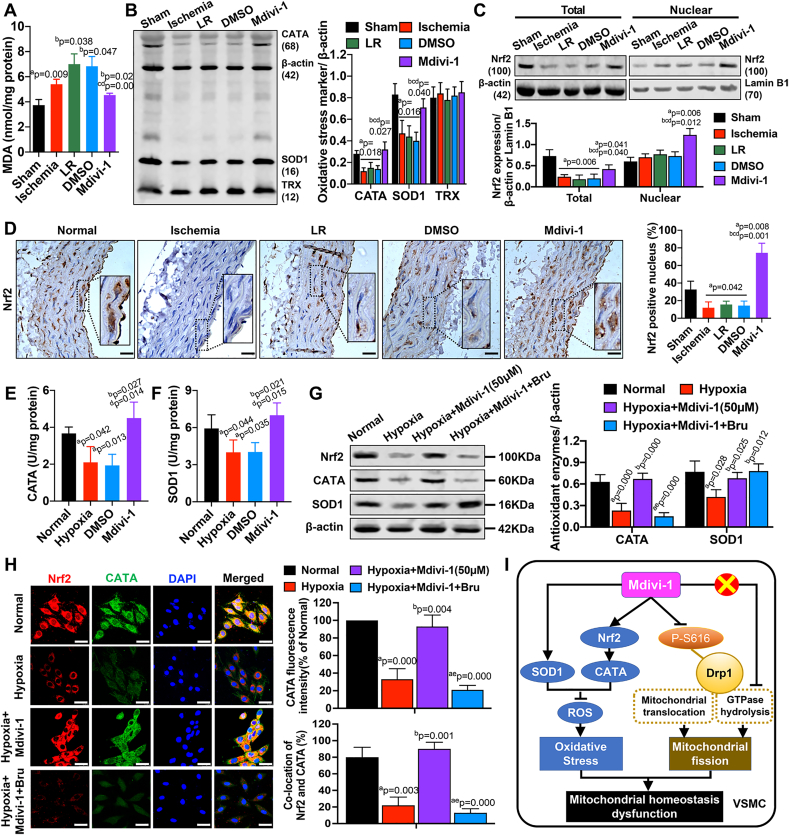
**Effects of Mdivi-1 on oxidative stress markers in vivo and in vitro**. (A)The level of malondialdehyde (MDA) in SMA tissues (n = 8 rats/group). (B) The protein expression of key antioxidant enzymes including catalase (CATA), superoxide dismutase 1(SOD1) and thioredoxin (TRX) in SMA tissues (n = 8 samples/group). (C) The total and nuclear expression of Nrf2 in SMA tissues (n = 8 samples/group). (D) The expression and distribution of Nrf2 in SMA tissues (n = 8 samples/group). (E) The level of catalase (CATA) in VSMCs (n = 8 samples/group). (F) The level of catalase (CATA) in VSMCs (n = 8 samples/group). (G) The protein expression of catalase (CATA) and superoxide dismutase 1(SOD1) in VSMCs (n = 8 samples/group). Bru = Brusatol, the Nrf2 inhibitor. (H) Confocal images showing the expression of Nrf2 and catalase (CATA) in VSMCs (n = 8 images/group). Bru = Brusatol. (I) Schematic diagram showing the possible signaling mechanisms of Mdivi-1 in hemorrhagic shock. a: P < 0.05 compared with Sham or Normal group, b: P < 0.05 compared with Shock or Hypoxia group, c: P < 0.05 compared with LR group, d: P < 0.05 compared with DMSO group, e: P < 0.05 compared with Hypoxia + Mdivi-1 group.

The content of malondialdehyde (MDA) was significantly increased after ischemia in SMA tissues. Infusion of LR further increased the MDA level after ischemia (P < 0.05), while Mdivi-1 administration significantly decreased MDA compared to the LR- and DMSO-treated rats (P < 0.05) (Fig. 6A), which further confirmed the inhibitory effects of Mdivi-1 on ischemia-induced oxidative stress in vessels. Moreover, ischemia caused a decrease in key antioxidant enzymes such as catalase (CATA) and superoxide dismutase 1 (SOD1), but not of thioredoxin (TRX), in SMA tissues. LR or DMSO did not affect the levels of these enzymes (P > 0.05). Notably, the infusion of Mdivi-1 significantly promoted the production of CATA and SOD1 in SMA tissues (P < 0.05) (Fig. 6B). Western blotting analysis showed that the total expression of NF-E2-related factor 2 (Nrf2) was significantly decreased after ischemia (P < 0.05). LR and DMSO did not influence Nrf2 expression (P > 0.05), while Mdivi-1 treatment caused an increase in the level of both total and nuclear Nrf2, compared to ischemic rats (P < 0.05) (Fig. 6C). Immunohistochemical analysis of SMAs further confirmed the effects of Mdivi-1 on nuclear Nrf2 expression *in vivo* (Fig. 6D). In VSMCs, a 4-h hypoxia caused a significant decrease in CATA and SOD1 levels, while treatment with Mdivi-1 attenuated oxidative stress by enhancing CATA and SOD1 levels after hypoxia (P < 0.05) (Fig. 6E–F), consistently with the above *in vivo* findings. These results suggested that the antioxidative effects of Mdivi-1 were likely due to the upregulation of antioxidant enzymes, as well as of Nrf2 expression, in VSMCs exposed to ischemic/hypoxic injury.

Furthermore, we found that promoting Nrf2 degradation by brusatol (Bru), an Nrf2 inhibitor, attenuated the Mdivi-1-induced increase in CATA level, while it had no effect on Mdivi-1-induced SOD1 upregulation (Fig. 6G). Confocal microscopy also showed that inhibiting Nrf2 could reduce CATA fluorescence increased by Mdivi-1 in hypoxia-treated VSMCs, which further confirmed the effect of Mdivi-1 on Nrf2/CATA-dependent oxidative pathway (Fig. 6H).

Taken together, our results demonstrated that Mdivi-1 protected vascular functions from ischemic/hypoxic injury by both attenuating oxidative stress and inhibiting excessive mitochondrial fission. On the one hand, Mdivi-1 significantly decreased ROS production and stimulated the antioxidant system in VSMCs by affecting SOD1-and Nrf2/CATA-related pathways in a Drp1-independent manner. On the other hand, Mdivi-1 caused a transient reduction of excessive mitochondrial fission by inhibiting Drp1 phosphorylation at Ser-616 at early stages of treatment, while it did not significantly affect Drp1 GTPase activity in VSMCs after ischemic/hypoxic injury (Fig. 6I).

### 3.6 The protective effects of Mdivi-1 on vascular functions stabilize hemodynamics, increase vital organ perfusions and improve survival outcomes after ischemic/hypoxic injury

**Fig. 7:**
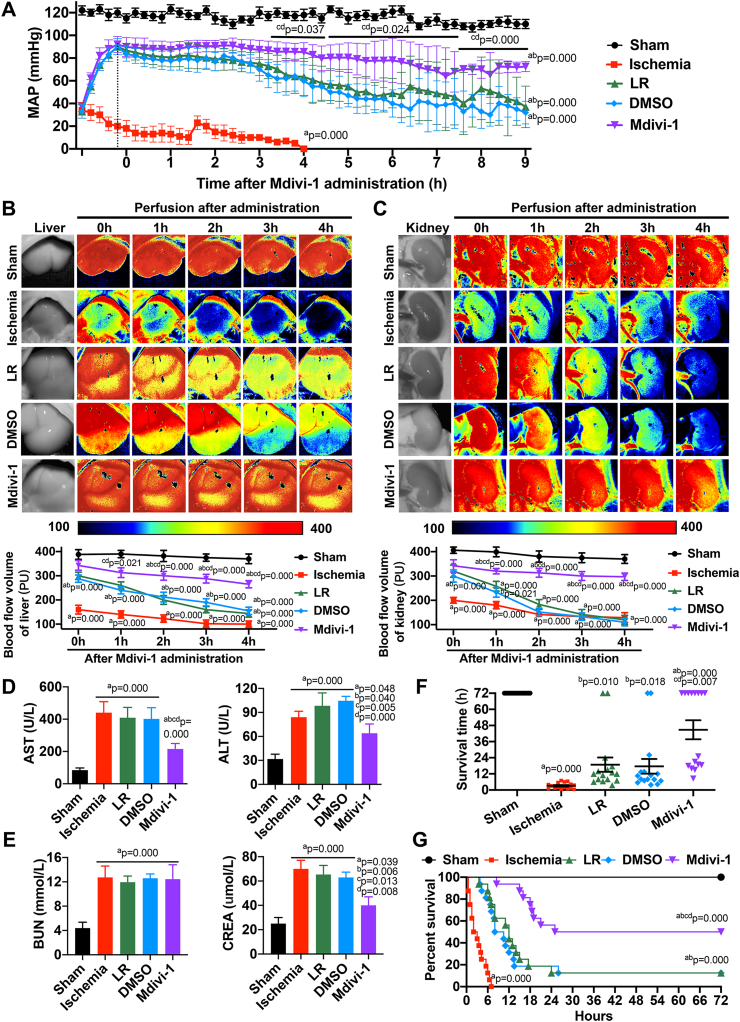
**Systemic effects of Mdivi-1 in ischemic/hypoxic injury**. (A) Ambulatory mean artery pressure (MAP) monitoring after administration. (n = 16 rats/group). (B) Time-lapse monitoring of liver blood flow using the laser speckle technique. (n = 8 rats/group). (C) Time-lapse monitoring of kidney blood flow using the laser speckle technique. (n = 8 rats/group). (D) Liver functions including aspartate aminotransferase (AST) and alanine aminotransferase (ALT). (n = 16 rats/group). (E) Kidney functions including blood urea nitrogen (BUN) and serum creatinine (CREA). (n = 16 rats/group). (F) Survival time after administration. (n = 16 rats/group). (G) Kaplan-Meier Survival curve.(n = 16 rats/group). a: P < 0.05 compared with Sham group at the same time point, b: P < 0.05 compared with Ischemia group at the same time point, c: P < 0.05 compared with LR group at the same time point, d: P < 0.05 compared with DMSO group at the same time point.

LR infusion after ischemia rapidly restored the mean artery pressure (MAP) from 30 mmHg to about 90 mmHg in rats (P < 0.05). However, at 3–4 h after resuscitation, the MAP began to decline in LR-treated rats (P < 0.05). At 6 h, while the MAP of Mdivi-1-treated rats was still above 80 mmHg, that of LR- and DMSO-treated rats had dropped to about 40 mmHg (P < 0.05) (Fig. 7A), suggesting that Mdivi-1 preserved vascular contraction, contributing to the maintenance of long-term blood pressure after resuscitation, and that exclusive LR treatment only had capacity expansion effect on ischemic/hypoxic injury.

Regarding the blood flow in vital organs such as liver and kidney, we observed significantly decreased perfusion after ischemia (P < 0.05). Two volumes of LR slightly and rapidly increased the blood flow but perfusion was hardly maintained after 2 h of resuscitation, especially in kidney (P < 0.05) (Fig. 7B–C). Moreover, liver and kidney functional parameters including AST, ALT, BUN, and CREA were significantly increased after ischemia. Mdivi-1 infusion significantly decreased the levels of AST, ALT, and CREA, as compared to the LR and DMSO groups (P < 0.05), whereas BUN was not affected by LR, DMSO, or Mdivi-1 (P > 0.05) (Fig. 7D–E).

We further investigated the effects of Mdivi-1 on rat survival after ischemic/hypoxic injury. In Mdivi-1-treated rats, the mean survival time and 72-h survival rate were 44.8 h and 50% (8/16), respectively, which were significantly higher than the corresponding values of ischemic (3.1 h, 0/16; P < 0.05), LR- (18.8 h, 2/16; P < 0.05) and DMSO-treated (17.6 h, 2/16; P < 0.05) rats (Fig. 7F). Death mainly occurred at 6–8 h and 15–20 h post-administration in LR- and Mdivi-1-treated rats, respectively (Fig. 7G), indicating that the protective effects of Mdivi-1 on vascular functions prolonged rat survival time and improved the survival rate after ischemic/hypoxic injury.

## 4 Discussion

The present study showed that Mdivi-1 had a beneficial effect on ischemic/hypoxic injury by improving vascular reactivity and VSMC contraction. Mdivi-1 is mainly used as a selective inhibitor of the Drp1 GTPase. Here we showed that, in VSMCs, Mdivi-1 antagonized ischemia-induced excessive mitochondrial fission and slightly enhanced mitochondrial fusion by reducing Ser-616-dependent Drp1 translocation to mitochondria, not by regulating Drp1 GTPase activity. Furthermore, Drp1 knockdown or general GTPase inhibition did not mimic the effects of Mdivi-1 on mitochondrial ROS levels after ischemia/hypoxia in vascular tissues or VSMCs, indicating that the protective action of Mdivi-1 was primarily attributable to Drp1-independent suppression of oxidative stress. Consequently, we concluded that the effects of Mdivi-1 on ischemic/hypoxic injury were not completely dependent on Drp1.

Drp1 is traditionally regarded as a mechano-chemical GTPase, and its GTPase hydrolysis is tightly regulated to ensure a strict control of mitochondrial size according to the cellular needs [23]. Mdivi-1 was first identified in 2008 via a chemical library screening, and found to inhibit the activity of the Dnm1 GTPase, the yeast homologue of mammalian Drp1 [9], likely by allosteric binding and subsequent inhibition of Dnm1 self-assembly. In recent years, several studies have explored Mdivi-1 mechanism of action in mammalian cells [13,24]. The available evidence in support or against a role of Mdivi-1 as an inhibitor of Drp1 GTPase is summarized by a commentary, which cites several in vitro studies demonstrating Mdivi-1-induced inhibition of Drp1 GTPase activity [25]. In the present study, the ability of Mdivi-1 to directly antagonize mammalian Drp1 GTPase activity under normal or hypoxia conditions was explored in VSMCs. The hydrolysis of Drp1-bound GTP was significantly increased after hypoxia, and Mdivi-1 failed to substantially affect Drp1 GTPase activity under both normal and hypoxic conditions. The most likely explanation for these findings is that Mdivi-1 may modulate Drp1 self-assembly rather than its GTPase activity in mammalian cells [13]. A previous study in rat neurons showed that Mdivi-1 only induced a ~25% decrease in Drp1-specific GTPase activity [13], which is consistent with the mild effect of Mdivi-1 on Drp1 GTPase activity that we observed in rat VSMCs. Moreover, a recent screening identified 17 compounds with high predicted affinity for the GTPase domain of Drp1, and evaluated their protective properties in an ischemia-reperfusion injury model [24]. In silico screening showed that various molecules, such as Drpitor1 and Drpitor1a, are more potent GTPase inhibitors compared to Mdivi-1. However, our study does not exclude the possibility that Mdivi-1 may indirectly influence Drp1 GTPase activity. Further investigation is needed to clarify this issue.

Our previous studies have shown that ischemia may cause vascular dysfunction by triggering Drp1-mediated mitochondrial fission [6,7]. In vascular tissues, during early stages of ischemic injury, Drp1 is activated at Ser-616 and subsequently translocated to the mitochondria, contributing to the regulation of mitochondrial dynamics [6]. We previously showed that ischemia induces various types of Drp1 modifications in intestinal tissues. These changes, including phosphorylation at Ser-616, dephosphorylation at Ser-637, as well as Drp1 de-ubiquitination and SUMOylation, are essential for the regulation of mitochondrial homeostasis [7]. However, the exact Mdivi-1 target site in Drp1 is still unclear. A study on pulmonary arterial hypertension showed that Mdivi-1 treatment in rats with right ventricular ischemia reduces Drp1 translocation to mitochondria and improves mitochondrial structure [26]. Moreover, Mdivi-1 was shown to abolish Drp1 dephosphorylation at Ser-637 in a rat model of cerebral ischemia [27], and to inhibit LPS-induced Drp1 phosphorylation at Ser-616, dephosphorylation at Ser-637, and translocation from the cytoplasm to mitochondria in a mouse model of sepsis [28]. In the present study, molecular docking assays and S616A mutagenesis allowed us to confirm a direct interaction between Mdivi-1 and Drp1 at Ser-616. The inhibitory effect of Mdivi-1 on ischemia-induced mitochondrial fission did not involve Drp1 Ser-637 in VSMCs. Moreover, we also observed a slight increase of mitochondrial fusion in hypoxia-treated VSMCs after early treatment with 50 μM Mdivi-1. A study of Bordt et al. showed that proteins involved in mitochondrial fusion, such as Mfn1, Mfn2, and Opa1, are significantly upregulated by Mdivi-1, suggesting that, in addition to inhibiting mitochondrial fission, Mdivi-1 may also influence mitochondrial fusion [13]. Hence, whether Mdivi-1 can regulate mitochondrial dynamics after ischemic/hypoxic injury in a Drp1-independent manner needs further investigation.

We here demonstrated that, in hypoxia-treated VSMCs, Mdivi-1 had a significant impact on mitochondrial dynamics only within 60 min of treatment, while the hypoxia-induced changes in mitochondrial fission and fusion rates, as well as mitochondrial fragmentation, were restored after 90–120 min. Previous studies showed that Mdivi-1 induces mitochondrial elongation within 1 h, while no effects on mitochondrial size were detectable with incubations longer than 5 h [9,13,29], in line with our conclusion that Mdivi-1 exerts a transient, short-term effect on mitochondrial dynamics and that, therefore, it may be suitable for early-stage treatment of ischemic/hypoxic injury.

The role of excessive ROS generation in oxidative stress and cardiovascular diseases is commonly accepted [30], and an appropriate balance between ROS and antioxidants is essential for vasoconstriction [31]. The effects of Mdivi-1 on oxidative stress are still controversial, although the antioxidative effects of Mdivi-1 are emphasized by most studies [28,32]. A slight increase of ROS level was observed in Mdivi-1-treated WT and Drp1 knockout mouse embryonic fibroblasts (MEFs) in the study by Bordt EA et al. [13]. We speculate that this might be a compensatory reaction to the moderate increase in ATP production because Mdivi-1 failed to stimulate ROS emission from isolated brain mitochondria in the absence of ADP [13]. Although the antioxidative effects of Mdivi-1 have been reported in several acute and severe disease conditions in recent years [33,34], the specific mechanism of action of this drug in the context of ischemic/hypoxic injury remains unclear. The current study showed that Mdivi-1 treatment following ischemic/hypoxic injury significantly decreased the levels of oxidative stress markers, such as MDA, and increased those of key antioxidant enzymes, such as SOD1 and CATA, in the vessels, via the Nrf2-dependent pathway. Nrf2 has been reported to regulate damage resistance and tissue remodeling by affecting various biological processes including autophagy, anabolic metabolism, extracellular matrix remodeling, and transcription [35]. Therefore, the effects of Mdivi-1 on Nrf2 expression or function, as well their impact on the regulation of mitochondrial homeostasis, need to be further elucidated.

The present study demonstrated that the antioxidative effects of Mdivi-1 in vascular tissues and VSMCs after ischemic/hypoxic injury were Drp1-independent. A study by Wang et al. showed that Mdivi-1 exerts Drp1-independent antimitotic effects and disrupts the cell cycle in both Drp1 wild-type and knockout mouse embryonic fibroblast cells [36]. Moreover, a recent study on brain ischemia reported that Mdivi-1 exerts Drp1-independent protective effects by modulating intracellular Ca^2+^ signaling and mitochondrial membrane depolarization [37,38]. These studies support our conclusion that the effects of Mdivi-1 on mitochondrial homeostasis are not dependent on Drp1 and further suggest that the mechanism of Mdivi-1-induced vascular protection after ischemic/hypoxic injury is more complex than previously believed [39,40].

## 5 Conclusions

Our results indicated that Mdivi-1 may exert remarkable protective effects against ischemic/hypoxic injury, improving animal survival and preserving vital organ perfusion. These effects were related to Mdivi-1-induced protection of vascular functions, hemodynamic stabilization, attenuation of oxidative stress, and improvement of mitochondrial dynamics. Nrf2 was involved in Drp1-independent Mdivi-1-mediated inhibition of oxidative stress. Mdivi-1 appeared to interfere with Drp1 phosphorylation at Ser-616, thus reducing Drp1 translocation to mitochondria after ischemia. Mdivi-1-induced inhibition of mitochondrial fission did not depend on its effect on Drp1 GTPase activity in VSMCs exposed to hypoxia.

The current study has some limitations. First, all experiments were performed in small animals, and further investigations are needed to confirm the anti-ischemic effects of Mdivi-1 in humans. Second, a model of severe ischemia (50% of total hemorrhage) was employed, and the effects of Mdivi-1 should be explored in less severe forms of ischemia or hemorrhage.

Notwithstanding the above, we here demonstrated that Mdivi-1 may be a promising therapeutic agent for the treatment of ischemic/hypoxic injury, especially at early stages.

## Funding

This work was supported by the 10.13039/501100001809National Natural Science Foundation of China (No. 81700429) and the Key Program of the National Natural Science Foundation of China (No. 81730059).

## Declaration of competing interest

The authors declare that they have no conflict of interests.

## Appendix A Supplementary data

Fig. S1 **The transfection and interference effects of GFP-tagged Adenovirus Scramble Drp1 (GFP-Ad-shDrp1)**. (A) VSMCs transfected with GFP-Ad-shDrp1. (B) Relative Drp1 expression in VSMCs after transfected with Ad-Vector, GFP-Ad-Vector and GFP-Ad-shDrp1.(n = 8 samples/group). Fig. S1
