# Supplementary figures and images for: Mdivi-1 attenuates oxidative stress and exerts vascular protection in ischemic/hypoxic injury by a mechanism independent of Drp1 GTPase activity
Source: Redox Biol. 2020 Aug 29;37:101706. doi: 10.1016/j.redox.2020.101706 (PMC7490562; doi:10.1016/j.redox.2020.101706)

**A**

## VSMCs transfected with GFP-Ad-shDrp1

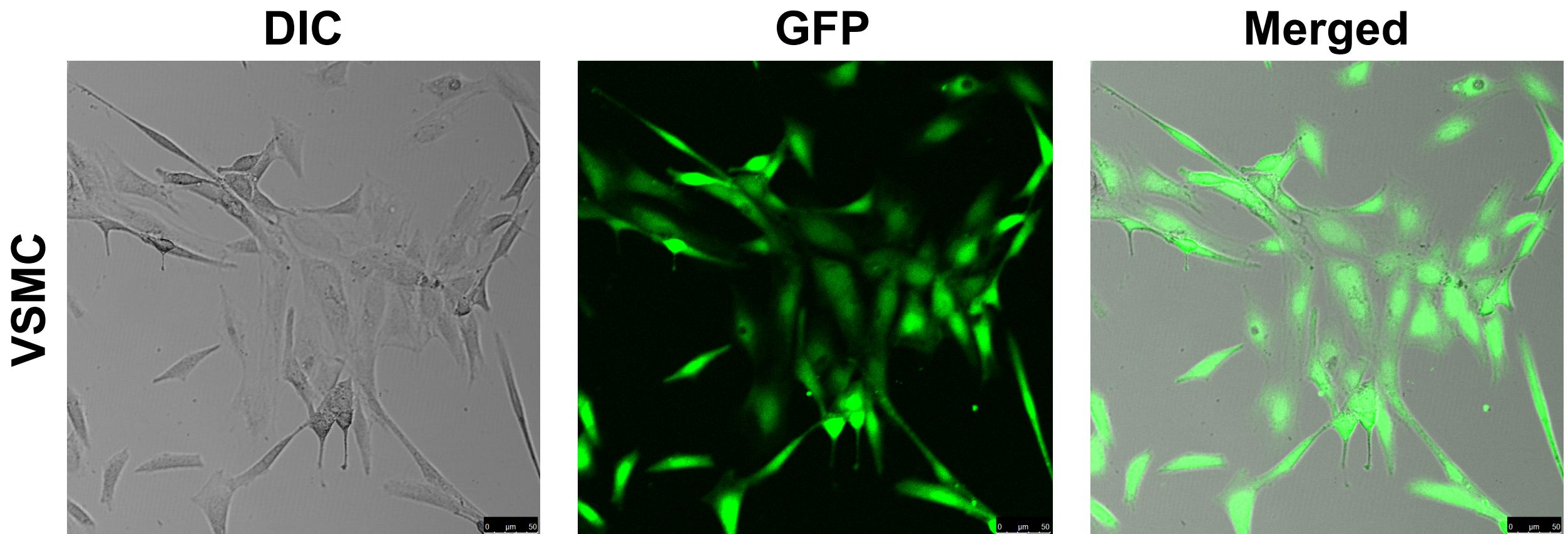**B**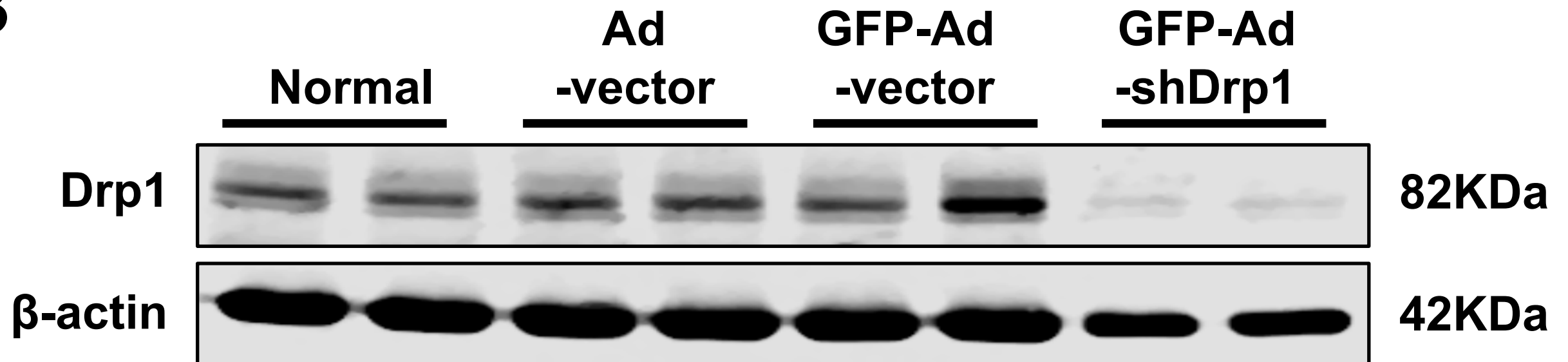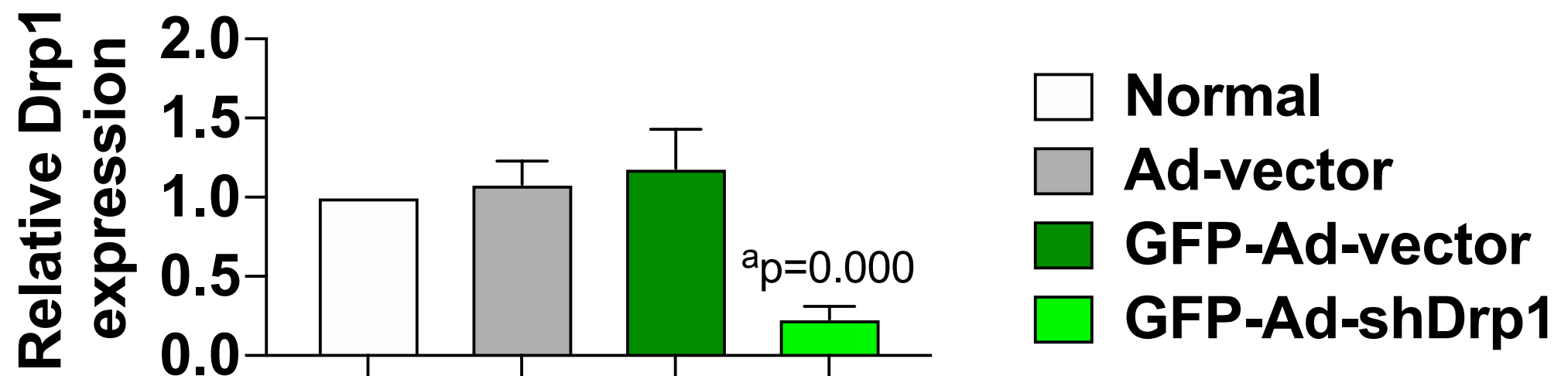

Supplement: Fig. S1 — The transfection and interference effects of GFP-tagged Adenovirus Scramble Drp1 (GFP-Ad-shDrp1). (A) VSMCs transfected with GFP-Ad-shDrp1. (B) Relative Drp1 expression in VSMCs after transfected with Ad-Vector, GFP-Ad-Vector and GFP-Ad-shDrp1.(n = 8 samples/group). [file mmc1.pdf]
